# Editorial: Current issues in sleep in children with neurodisability

**DOI:** 10.3389/frsle.2023.1317714

**Published:** 2023-10-31

**Authors:** Jasneek Chawla, Laurie McLay, Moya Vandeleur

**Affiliations:** ^1^Department of Respiratory and Sleep Medicine, Queensland Children's Hospital, Brisbane, QLD, Australia; ^2^Child Health Research Centre, Faculty of Medicine, The University of Queensland, Brisbane, QLD, Australia; ^3^Faculty of Health, The University of Canterbury, Christchurch, New Zealand; ^4^Child Well-being Research Institute, University of Canterbury, Christchurch, New Zealand; ^5^Department of Respiratory and Sleep Medicine, The Royal Children's Hospital, Melbourne, VIC, Australia; ^6^Respiratory Group-Infection, Immunity and Global Health Theme, Murdoch Children's Research Institute, Melbourne, VIC, Australia

**Keywords:** sleep, neurodisability, pediatric, children, health

It is increasingly recognized that sleep is an important factor that requires attention in children with neurodisability (ND). Existing literature suggests that sleep disorders can affect as many as 80% of children in this population (McDonald and Joseph, 2019). Yet surprisingly this is a relatively understudied field, with no published clinician-focused guidelines specific to management of sleep problems in children with ND. Rather, the approach to management is to extrapolate from the methods used in neurotypical children, relying on the individual clinician's experience to modify and adapt management to suit the needs of a particular child. In this series we collate international research, relating to sleep problems in children with ND, aiming to highlight the breadth of research being undertaken in this field and helping to reduce the knowledge gap relating to sleep in children with ND. Original research undertaken by experienced researchers, using a range of methodologies is presented, covering diverse topics relating to all aspects of sleep disorders encountered in children with ND.

McPhee et al. draw upon data from three large Canadian databases, to examine the rates of sleep disturbances in children with and without neurodevelopmental disabilities. The study demonstrates how pooled samples from registries can be utilized for secondary data analyses to address important knowledge gaps relating to distinct clinical populations, such as children with ND. Additionally, it provides an impetus for the pediatric sleep community to advocate for sleep parameters to be included routinely in the data collected in such registries by highlighting once again the greater proportion of children with ND who experience sleep disturbance compared to children with TD.

In their article, Bruce et al. explored parents' perspectives on the role of exercise in autistic children, including its effects on sleep. Contrary to the existing body of research demonstrating the benefits of exercise on sleep (Buman and King, 2010), mothers in this study reported both positive and negative effects, raising concerns around overstimulation, which then delayed sleep. Such findings are invaluable to clinicians working with children with ND. They can help to explain why strategies for improving sleep should be modified from those used in TD populations, rather than directly adopted, to consider the impact of the individual phenotype of a specific child or population. The results of this study demonstrate how qualitative research can provide additive information to that of quantitative studies.

This point is further illustrated in two articles in this Research Topic from the Canadian team who have developed the *Better Nights, Better Days*^1^ sleep intervention (BNBD) for insomnia in children. The authors of these studies describe how engagement with those with lived experience can assist in the development and implementation of sleep supports for this population. Jemcov et al. provide an insightful short report from exit interviews undertaken with fifteen parents of children aged 4–10 years with ND, who trialed the Better Nights, Better Days intervention for typically developing children (BNBD-TD). Data were collected to inform the development of Better Nights, Better Days intervention for children with neurodevelopmental disorders (BNBD-NDD). In the next phase of this work, Ilie et al. describe the experiences of twenty parents who implemented the modified BNBD-NDD program during the randomized controlled trial evaluation of this intervention (Corkum et al., 2018). Both of these studies identify how, despite motivation, some families of children with ND may require additional supports to implement strategies to improve sleep. This suggests that to be effective, the approach to implementing an intervention for sleep in children with ND, needs to not only be tailored to this population, but also needs to provide methods that can overcome the additional stressors and demands already faced by families caring for a child with ND. This may include for example, more regular check ins with parents and achievable goal setting to facilitate improved outcomes for sleep in children with ND.

The need for additional support for families is also implied in the article by Valji et al. Whilst the focus of this article is on demonstrating how children with Down syndrome (DS) with upper airway obstruction can be successfully managed with long-term non-invasive support (LT-NIV), this retrospective study in a relatively large cohort of children, did demonstrate lower adherence to therapy in children with DS when compared to matched controls. The authors suggest exploring potential factors that may influence adherence to therapy at an early stage may be important. Drawing parallels between the findings of this study and that of the Better Days, Better Nights team, one of the approaches that could potentially be beneficial for children with DS and other ND conditions who require NIV may be to provide families with more intensive support during the initiation phase of therapy. Further research is required to explore the effectiveness of this, and other methods that may provide targeted support for families, during the implementation phase of new management strategies for children with ND. As this Research Topic highlights, the importance of engaging with primary caregivers and obtaining insights on their experiences is essential and cannot be underestimated.

## Author contributions

JC: Writing – original draft. LM: Writing – review & editing. MV: Writing – review & editing.

## Footnotes

^1^ https://betternightsbetterdays.ca
